# A high density of ultra-processed food, alcohol & tobacco retail stores, and social inequalities are associated with higher mortality rates of non-communicable diseases in Mexican adults: 2005 to 2021

**DOI:** 10.1371/journal.pone.0301387

**Published:** 2024-04-10

**Authors:** Adriana Garduño-Alanis, Alejandra Contreras-Manzano, Juan Carlos Salgado, Héctor Lamadrid-Figueroa, Katherine Curi-Quinto, Simón Barquera

**Affiliations:** 1 Center for Nutrition and Health Research, National Institute of Public Health, Cuernavaca, Morelos, Mexico; 2 National Council of Humanities, Science and Technology, Mexico; 3 Autonomous University of the State of Mexico, Toluca, State of Mexico, Mexico; 4 Center for Population Health Research, National Institute of Public Health, Cuernavaca, Morelos, Mexico; 5 Instituto de Investigación Nutricional, Lima, Peru; Facultad Latinoamericana de Ciencias Sociales Mexico, MEXICO

## Abstract

**Background:**

Non-communicable diseases (NCDs) are the leading causes of mortality in Mexico. Factors contributing to NCDs-related deaths may vary across small geographic areas such as municipalities. We aimed to predict municipal-level factors associated with NCD mortality in Mexican adults from 2005 to 2021 using the small-area analysis (SSA) approach.

**Methods:**

We gathered data on population sociodemographic, access to healthcare services, and mortality records at the municipal-level from census and public institutions from 2005 to 2021. We identified municipal predictors of NCDs mortality rates (MR) using negative binomial regression models.

**Results:**

A total of 584,052 observations of Mexican adults were analyzed. The national expected NCDs MR per 100,000 inhabitants was 210.7 (95%CI: 196.1–226.7) in 2005 and increased to 322.4 (95%CI: 300.3–346.4) by 2021. Predictors of NCDs mortality (quintile 5 vs. quintile 1) included; indigeneity (IRR = 1.15, 95%CI: 1.12–1.19), poverty (IRR = 1.14, 95%CI: 1.13–1.15), affiliation with Mexican Social Security Institute (IRR = 1.11, 95%CI: 1.09–1.14), households with television (IRR = 1.14, 95%CI: 1.11–1.17), and high density of ultra-processed food, alcohol & tobacco retail stores (IRR = 1.15, 95%CI: 1.13–1.17). The greatest increases in MR were observed in municipalities from Oaxaca (>200% increments).

**Conclusion:**

There was an overall increase in NCDs MR from 2005 to 2021, with a significant geographic variation among Mexican municipalities. The results of this study highlight the importance of identifying priority areas in the country that urgently require public policies focused on local factors associated with deaths from NCDs, such as the regulation of the ultra-processed food, alcohol & tobacco retail stores, and efforts to reduce social inequalities.

## Introduction

Non-communicable diseases (NCDs) encompass a range of health conditions, including type 2 diabetes (T2D), cardiovascular diseases (CVD), and chronic kidney disease (CKD) [1, 2]. These NCDs often share common risk factors such as hypertension, dyslipidemia, and obesity. In 2019, CVDs accounted for approximately 17.9 million global deaths, representing 32% of all mortality. Additionally, T2D, which includes CKD-related mortality, contributed to about 2 million deaths [1]. Notably, ischemic heart disease and stroke emerge as leading causes of disability-adjusted life years in the adult population [3].

NCDs represent a significant health challenge due to their high rates of morbidity and mortality [1]. The health of a population is influenced by both, individual characteristics and social determinants, such as, ethnicity, education, access to healthcare, socioeconomic status, and environmental factors including food availability [1, 4, 5]. These health and social determinants can vary significantly across different geographical regions. For example, Mexico, with 2,454 municipalities spread across 32 states, each characterized by diverse culture, population densities, economies and geographic extension, all of which can impact the burden of NCDs [5, 6]. Hence, it becomes relevant to provide meaningful evidence of the relationship between community-level factors and NCDs mortality. The small-area analysis (SAA) approach offers a valuable tool for examining geographic disparities and their associations with health outcomes, providing essential insights into community-level factors [7]. Moreover, this approach is globally employed and plays an essential role in assessing the influence of small-area factors on mortality rates [7].

The government prioritizes interventions and public policies aimed at addressing the growing burden of NCDs [8]. In Mexico, NCDs have consistently ranked among the top ten causes of death for the past two decades, accounting for nearly 54% of deaths in 2005 and approximately 60% in 2021, excluding COVID-19 deaths [6]. This scenario underscores the profound impact of NCDs on the overall health of the population, emphasizing the urgent need for prevention and mitigation efforts [8]. By identifying the factors at the municipality-level that predict NCDs mortality, we can equip policymakers with valuable insights. This information can help tailor public policies and allocate resources more effectively for the prevention and control these diseases [5, 8]. Therefore, the objective of this study was to identify the municipal-level factors associated with NCDs mortality in Mexican adults from 2005 to 2021 using the SAA approach [9].

## Methods

### Study design

We conducted an ecological study using municipal-level data for Mexican adults from 2005 to 2021 to identify heterogeneity within large geographic areas by examining homogeneous sub-areas. Small-area estimation is a statistical approach employed to predict characteristics of interest, such as mortality rates, utilizing existing data. This method involves substantial stratification based on geographic and temporal factors, especially for small geographical units. By avoiding the need for primary data collection within each unit, it streamlines the estimation process [7]. In the context of Mexico, the smallest geographical and administrative units are the municipalities, whose areas range from 2.2 to 53,104 Km^2^, and population density ranges from 0.14 to 19,436 inhabitants/ Km^2^ [10]. Adopting the municipality as the small-area unit for our analysis, we used available data on population sociodemographics, access to healthcare services, and mortality records at the municipal level. This data, sourced from censuses and public institutions covering the period from 2005 to 2021, facilitated the estimation of municipal predictors for NCDs mortality rates, stratified by sex and quinquennium of age. Following the SSA approach, we initially designated the municipality as the primary area of interest, encompassing a total of 2,454 municipalities in Mexico for the year 2005. We processed microdata from 2005 to 2021 and fitted a negative binomial model to estimate the relationship between observed municipal factors and NCDs mortality. Subsequently, we predicted the standardized MR for each municipality in each year. Additionally, we generated annual state-level graphs and municipal-level maps based on both crude and standardized MRs.

### Data sources

We collected data from official public open-access microdata at the municipal-level from 2005 to 2021. The municipal-level was defined in accordance with Mexico’s 2005 geographical division encompassing a total of 2,454 municipalities in 32 states [6]. Databases were obtained from the National Population Council (CONAPO) [11], National Autonomous University of Mexico (UNAM) [10], National Institute of Statistics and Geography (INEGI) [6], National Council for the Evaluation of Social Development Policy (CONEVAL) [12], National Directory of Economic Units (DENUE) [6], and the General Direction of Health Information (DGIS) of the Ministry of Health [13].

### Study outcome

Our dependent variable was the number of deaths from NCDs. The single variable that encompassed the total deaths from NCDs included deaths from: T2D, CVD, CKD, cardiovascular diseases (CVD), hypertension, dyslipidemia and obesity. The data pertaining to adult deaths (individuals aged ≥20 years) attributed to NCDs were obtained from DGIS [13] for the period spanning from 2005 to 2021. The NCDs considered in this study were categorized in accordance with the International Statistical Classification of Diseases, 10th edition (ICD-10). Our dependent variable was a composite variable encompassing the total deaths from NCDs, including T2D (codes E10-E14), CVD (codes I20-I25 and I63-I64), and CKD (codes N18 and N19), along with deaths related to the following NCD risk factors: hypertension (codes I10, I15, O10, R030), dyslipidemia (code E78), and obesity (code E66). These variables were standardized as annual municipal rates, stratified by sex and quinquennium of age.

### Predictor variables

We employed several sociodemographic and access to healthcare services predictor variables derived from diverse sources to comprehensively assess municipal-level factors. The predictor variables were selected for being available at the municipal level and being considered factors associated with a higher mortality risk from NCDs at the individual level [6, 14–24]. In the negative binomial model, the cases of deaths due to NCDs per year, municipality, age group, and gender were the dependent variable. The analysis units consisted of age and sex categories within the 2,454 municipalities per year Sociodemographic and municipal environmental predictor variables were transformed into annual proportions, densities, and rates per 100,000 inhabitants. The exposure in the model was the adult population of the municipality per year, age group, and gender, allowing for the adjustment of mortality rates based on the population. The population size of municipalities was based on CONAPO projections [11]. Municipal geographic area (in square kilometers) was obtained from estimations provided by UNAM to estimate population density (Total population/Km^2^) [10, 14, 15]. We collected demographic information, including the proportions of indigenous speakers aged ≥5 years, illiteracy in population aged ≥15 years, from INEGI counts (2005) and census data (2010 and 2020) [6, 22, 23]. Information on the population affiliated with social security services and proportion of households with television was also obtained from INEGI [6, 16, 24]. Poverty estimations at the municipal level were retrieved for the years 2010, 2015 and 2020 from CONEVAL, a governmental institution that assesses and classifies poverty according to income and the presence of the following social deprivations: education, healthcare, social security, housing services, or access to food. The three categories of poverty from CONEVAL’s evaluation are: a) Poverty: population whose income is below the value of the well-being line and who experiences at least one social deprivation, b) Extreme poverty: population experiencing three or more social deprivations and whose income is below the minimum well-being line, c) Moderate poverty: it refers to individuals who, while being poor, do not fall into the category of extreme poverty. The incidence of moderate poverty is calculated by determining the difference between the incidence of the population in poverty and that of the population in extreme poverty. For our study, we used estimations of moderate poverty at the municipal level [12, 18]. We evaluated the ultra-processed food, alcohol & tobacco retail environment at municipal-level using microdata from INEGI, which has been annually registering economic units’ activities, size (base on employed personnel), and locations in the country since 2010 [6, 17]. Each economic units in the DENUE database was coded according to the criteria of the North American Industrial Classification System (NAICS) criteria [6, 25]. We characterized the ultra-processed food, alcohol & tobacco retail environment using codes associated with higher households purchases of ultra-processed-products (UPP) reported by Hernández M, et al. [25] that includes convenience stores, discount stores, small grocery stores, and stores specialized in selling candies, popsicles, soft drinks, and other food and beverages. Additionally, codes for stores selling alcohol & tobacco were incorporated into the classification of ultra-processed food, alcohol & tobacco retail environment. To assess the ultra-processed food, alcohol & tobacco retail environment, we calculated the store density by dividing the number of stores by the total population per 100,000 inhabitants at the municipal level.

### Statistical methods

To address missing values in predictor variables not measured between census years (2006–2009, 2011–2019, and 2021), we employed linear and logistic regression models adjusted by age and sex. To standardize the predictor variables, we transformed them into proportions, densities, or rates per 100,000 inhabitants, using population projections from CONAPO at the municipal level, stratified by age group and sex as the denominator. Furthermore, to examine potential non-linear associations, we categorized predictors variables into quintiles over the study period. To estimate the municipal factors associated with the NCDs MR, we utilized negative binomial regression models. These models were adjusted for sex, age group, predictor variables (categorized in quintiles) and year. The dependent variable consisted of the number to NCDs-related deaths by year, sex, age group, and municipality of residence. We assessed multicollinearity among covariates using the diagnostic tool of Variance Inflation Factor (VIF), incorporating variables with values <5 in the statistical models [26]. The final model selection was guided by achieving the best goodness of fit, determined by the Akaike Information Criterion (AIC) and the Bayesian Information Criterion (BIC). We adjusted the models using predictor variables in both continuous and categorical formats. The expected MR per 100,000 inhabitants was calculated using the predicted deaths from the negative binomial regression model and was subsequently age-standardized according to Mexico’s 2015 age structure. Additionally, recognizing the impact of excess mortality during the COVID-19 pandemic [27], we conducted a separate model to analyze municipal-level factors from 2005 to 2019. All analyses and maps were developed with Stata 17 (College Station, TX).

Ethics committee approval was not required for this study which analyzed publicly available datasets. Patient consent for publication was not required.

Equation of the model:

$$log\;\left(\frac{{NCDs\;deaths}_{jt}}{n_{jt}}\right)=\beta_{0}+\;\sum_{i=1}^{i=2}\beta_{1i\;}\left(D=i\;\right|\;\chi_{1jt})\;+\;\sum_{i=2}^{i=5}\beta_{2i\;}\;\left(D=i\;\right|\;\chi_{2jt})\;+\cdots +\;\sum_{i=2}^{i=32}\beta_{16i}\;\left(D=i\;\right|\;\chi_{16jt})\;+\mu_{j}+\;\varepsilon_{jt}$$

Where:

NCDs deaths: Mortality from NCDs in adults ≥20 years old (Dependent variable)

j: municipality

t: year (2005–2021)

n: inhabitants in the municipality ≥20 years old, by age group and gender.

β_0_: intercept

D = i | χ_1jt_: D stands for a dummy variable that equals one for category i in χ_1jt_. The first category in χ_1jt_ is the reference group.

Covariates: χ_1jt_: sex, χ_2jt_: age group (quinquennium in adults ≥20 years), χ_3jt_: proportion of males, χ_4jt_: proportion of indigenous population, χ_5jt_: proportion of illiteracy, χ_6jt_: proportion of moderate poverty, χ_7jt_: population density, χ_8jt_: density of ultra-processed food, alcohol & tobacco retail stores, χ_9jt_: proportion of households with television, χ_10jt_: density of hospitals, χ_11jt_: rate of hospital discharges from NCDs, χ_12jt_: proportion of the population affiliated to Mexican Social Security Institute, χ_13jt_: proportion of the population without social security, χ_14jt_: crude mortality rate from causes other than NCDs, χ_15jt_: year, χ_16jt_: state of the country.

i: covariates categories (e.g., i = (0,1,2) for the sex variable as in χ_1jt_ or i = (1,2,3,4,5) for the quitile-based variable as in χ_2jt_)

ε: unobserved random variables (or error term)

μ: unobserved fixed variables

## Results

A total of 584,052 observations of Mexican adults were analyzed. Table 1 shows municipal characteristics across the relevant study years. On average, among the 2,454 municipalities from 2005 to 2021, proportions of indigeneity, illiteracy, unaffiliated with health services and the density of ultra-processed food, alcohol & tobacco retail stores tended to decrease, while proportions of the population in poverty condition, households with television, and affiliated with Mexican Social Security Institute (IMSS, by its acronym in Spanish) showed an upward trend. Table 2 displays the crude and expected MR for NCDs at the national-level. In 2005, there were 219 NCDs-related deaths per 100,000 Mexican adults, increasing to 396 cases by 2021. The standardized MR for 2005 was predicted to be 210.7 (95%CI: 196.1–226.7) and increased to 322.4 (95% CI: 300.3–346.4) by 2021.

**Table 1:** Municipal characteristics in Mexico across the years 2005, 2010, 2015, and 2021.

	Year	**2005**	**2010**	**2015**	**2021**
		**Count**	**Census**	**Estimated**	**Estimated**
		**n (thousands)**	**n (thousands)**	**n (thousands)**	**n (thousands)**
**Population** ^1^ **(≥20 years old)**	Overall	61,397	69,058	76,818	85,080
	Females	32,054	36,050	40,026	44,269
	Males	29,343	33,008	36,792	40,811
		**Mean** **(95% CI)**	**Mean** **(95% CI)**	**Mean** **(95% CI)**	**Mean** **(95% CI)**
**Population density** ^1^^,^^2^	Total Population / km^2^	265 (219, 312)	282 (234, 329)	298 (249, 356)	313 (264, 363)
**Sociodemographic factors**		**%** **(CI 95%)**	**%** **(CI 95%)**	**%** **(CI 95%)**	**%** **(CI 95%)**
**Sex** ^1^^,^^3^	Male adults	47.8 (47.7, 47.8)	47.7 (47.6, 47.8)	47.8 (47.7, 47.9)	47.9 (47.8, 48)
**Indigeneity** ^a^^,^^1^^,^^3^ **(≥5 years old)**	Overall	19.6 (18.3, 20.8)	19.4 (18.1, 20.6)	18.7 (17.5, 19.9)	18.2 (17, 19.3)
	Females	18.3 (17.1, 19.4)	19.4 (18.1, 20.6)	18.6 (17.4, 48)	18.4 (17.2, 19.6)
	Males	20.5 (19.2, 21.8)	19.4 (18.1, 20.6)	19.1 (17.9, 20.3)	17.1 (16, 18.2)
**Illiteracy** ^a^^1^^,^^3^ **(≥15 years old)**	Overall	16.7 (16.3, 17.1)	14 (13.6, 14.4)	12 (11.6, 12.3)	9.8 (9.5, 10.1)
	Females	10.3 (10, 10.6)	8.6 (8.4, 8.9)	7.3 (7.1, 19.3)	6 (5.8, 6.2)
	Males	6.4 (6.2, 6.6)	5.4 (5.2, 5.5)	4.6 (4.5, 4.7)	3.8 (3.7, 4)
**Poverty** ^b^^,^^1^^,^^4^	Overall	41.2 (40.7, 41.7)	41.5 (41.1, 41.9)	45.8 (45.3, 46.3)	46.0 (45.5, 46.5)
**Density of ultra-processed food, alcohol & tobacco retail stores** ^c^^,^^1^^,^^5^	(n stores / Total population) * 100,000 hab.	904 (874, 934)	846 (823, 868)	817 (797, 836)	823 (804, 843)
**Households with television** ^a^ ^,^ ^3^	Inhabitated	74.9 (74.1, 75.8)	80.5 (79.8, 81.1)	80.0 (79.4, 80.7)	82.0 (81.4, 82.6)
**Healthcare Services Access**		**Rate or proportions** **(95% CI)**	**Rate or proportions** **(95% CI)**	**Rate or proportions** **(95% CI)**	**Rate or proportions** **(95% CI)**
**Hospital density** ^1^ ^,^ ^6^	(hospitals/Total population) * 100,000 hab.	2.4 (2.2, 2.6)	2.3 (2.1, 2.5)	2.3 (2.1, 2.5)	2.4 (2.3, 2.6)
**Affiliated with IMSS** ^a^^,^^1^^,^^3^	Total	14.5 (13.8, 15.1)	16.1 (15.5, 16.7)	15.9 (15.3, 16.6)	18 (17.3, 18.7)
**Unaffiliated with health services** ^a^^,^^1^^,^^3^	Total	68.2 (67.3, 69.1)	37.7 (37, 38.4)	34.1 (33.6, 34.6)	19.4 (19, 19.8)

^1^CONAPO: National Population Council.

^2^UNAM: National Autonomous University of Mexico.

^3^INEGI: National Institute of Statistics and Geography.

^4^CONEVAL: National Council for the Evaluation of Social Development Policy.

^5^DENUES: National Directory of Economic Units.

^6^DGIS: General Direction of Health Information of the Ministry of Health. IMSS: Mexican Social Security Institute.

^a^ Data for years 2006–2009, 2011–2019 and 2021 were estimated for each municipality through year-adjusted logarithmic regressions.

^b^ Data for years 2005–2009, 2011–2014 and 2016–2019 were estimated for each municipality through year-adjusted logarithmic regressions.

^c^ Data for years 2005–2010 were estimated for each municipality through year-adjusted logarithmic regressions.

**Table 2:** National mortality rates per 100,000 inhabitants in Mexican adults for non-communicable chronic diseases: Overall and by sex (2005–2021).

Year	Population Thousands	Deaths Cases	Crude Mortality Rate			Standardized mortality rate ^a^		
			Overall	Males	Females	Overall	Males	Females
			Rate	Rate	Rate	Rate (95% CI)	Rate (95% CI)	Rate (95% CI)
2005	61400	134433	219	229.2	209.6	210.7 (196.1, 226.7)	243.6 (226.4, 262.4)	235 (215, 257.2)
2006	62800	130644	207.9	220.7	196.2	197.7 (184, 212.8)	231.8 (215.4, 249.6)	217.2 (198.7, 237.7)
2007	64300	132398	205.8	219.7	193	194.2 (180.7, 208.9)	228.1 (212, 245.5)	211.7 (193.7, 231.7)
2008	65900	143011	216.1	230.7	202.7	202.5 (188.5, 217.9)	237.4 (220.7, 255.5)	220.3 (201.6, 241.1)
2009	67500	151343	224.3	240.4	209.6	208.2 (193.8, 224)	244.9 (227.7, 263.5)	225.4 (206.4, 246.7)
2010	69100	165923	239.3	257.1	223	220.6 (205.4, 237.2)	259.9 (241.8, 279.6)	237.1 (217.2, 259.3)
2011	70600	163028	230.8	248.9	214.1	210.4 (195.8, 226.2)	249.2 (231.9, 268.1)	225.1 (206.1, 246.2)
2012	72200	178691	247.4	266.1	230.2	223.5 (208.1, 240.3)	264.1 (245.7, 284)	239.6 (219.4, 261.9)
2013	73800	189102	256.3	276.4	237.8	229.2 (213.4, 246.4)	271.3 (252.5, 291.8)	244.5 (224, 267.3)
2014	75300	198028	262.9	284.1	243.4	232.7 (216.7, 250.1)	276 (256.9, 296.8)	247.3 (226.6, 270.4)
2015	76800	208776	271.8	292.9	252.3	238.5 (222.1, 256.3)	281.7 (262.2, 302.8)	253.6 (232.4, 277.1)
2016	78300	225655	288.3	312.3	266.2	249.7 (232.6, 268.3)	297 (276.5, 319.2)	263.9 (241.9, 288.4)
2017	79700	230426	289.2	313.6	266.8	247.8 (230.8, 266.3)	294.6 (274.3, 316.7)	261.4 (239.5, 285.7)
2018	81100	233138	287.6	314	263.3	242.6 (226, 260.8)	291.2 (271.1, 313)	253.3 (232.1, 276.9)
2019	82400	242384	294.1	321.7	268.6	245.7 (228.9, 264)	294.5 (274.2, 316.5)	256 (234.7, 279.8)
2020	83800	353019	421.5	481.4	366.3	344.2 (320.6, 370)	433.3 (403.5, 465.8)	341.8 (313.2, 373.6)
2021	85100	336958	396	442.2	353.5	322.4 (300.3, 346.4)	392.9 (365.9, 422.2)	327.5 (300.2, 357.9)

^a^ Estimates from binomial regression model adjusted by municipal structure of age, sex, and quintiles of the distribution of male proportion, indigeneity, illiteracy, poverty, population density, density of ultra-processed food, alcohol & tobacco retail stores, households with television, density of hospitals, affiliated with Mexican Institute of Social Security, unaffiliated with health services, mortality rate from causes other than non-communicable diseases, year, and state of the country. Predicted mortality rate was standardized by using the structure of age of 2015 for Mexican adults aged 20 years, from CONAPO.

**Fig 1:**
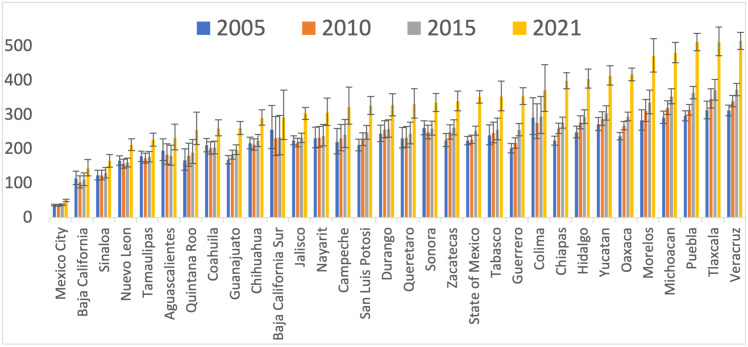
Non-communicable diseases standardized mortality rate per 100,000 Mexican adults from 2005 to 2021. Estimates from binomial regression model adjusted by municipal structure of age, sex, and quintiles of the distribution of male proportion, indigeneity, illiteracy, poverty, population density, density of ultra-processed food, alcohol & tobacco retail stores, households with television, density of hospitals, affiliated with Mexican Institute of Social Security, unaffiliated with health services, mortality rate from causes other than non-communicable diseases, year, and state of the country. Predicted mortality rate was standardized by using the structure of age of 2015 for Mexican adults aged 20 years, from CONAPO.

Fig 1 shows the standardized NCDs MR per 100,000 Mexican adults across States (2005–2021). The highest increases in MR were estimated for Guerrero, Oaxaca, and Chiapas, with increments of 75%, 75.8%, and 77.7%, respectively. Conversely, the lowest estimated increases were in Baja California Sur, Aguascalientes, and Coahuila, with increments of 13.4%, 18.7%, and 24%, respectively. Mexico City, Baja California and Sinaloa remained with lowest MR in the study period.

**Fig 2:**
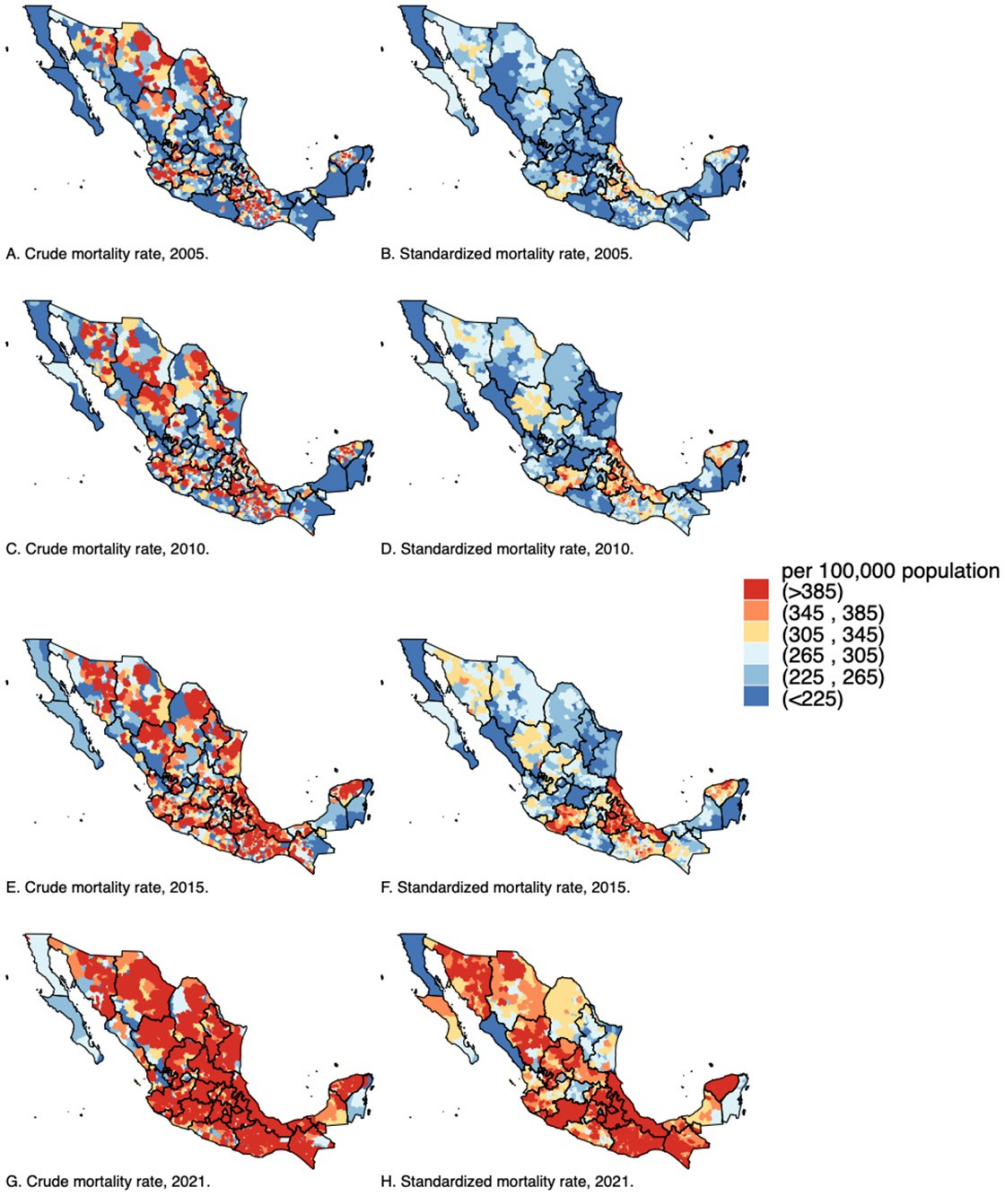
Non-communicable diseases geographic distribution of crude and standardized mortality rate per 100,000 Mexican adults from 2005 to 2021. Estimates from binomial regression model adjusted by municipal structure of age, sex, and quintiles of the distribution of male proportion, indigeneity, illiteracy, poverty, population density, density of ultra-processed food, alcohol & tobacco retail stores, households with television, density of hospitals, affiliated with Mexican Institute of Social Security, unaffiliated with health services, mortality rate from causes other than non-communicable diseases, year, and state of the country. Predicted mortality rate was standardized by using the structure of age of 2015 for Mexican adults aged 20 years, from CONAPO.

Fig 2 shows the geographic distribution of crude and expected NCDs MR per 100,000 inhabitants. We observed heterogeneous MR in most municipalities of the country in the specified years. Both crude and standardized MR remained elevated in the Gulf Coast, Central and Southeastern regions. In the north, there was a slight increase, while the South presented a more substantial rise.

The municipalities with the highest percentage increase in the MR were San Andrés Nuxiño and San Juan Achiutla, both in Oaxaca, and Nicolás Ruíz in Chiapas, with increases of 201.6%, 200.7%, and 191.6%, respectively. Conversely, the municipalities with the lowest percentage increase during the same period were Salinas Victoria and Marín, both in Nuevo León, and Ramos Arizpe in Coahuila, with increases of 1.2%, 3.8%, and 6.4%, respectively. Out of the 2,454 municipalities, the top 100 with the highest expected NCDs MR (ranging between 569 and 676 per 100,000 inhabitants) were from: Puebla (43%), Veracruz (22%), Oaxaca (21%), Tlaxcala (8%), and Michoacan (5%), (S1 Table). When comparing to the rest of the municipalities, the 100 municipalities had a significantly higher density of unhealthy product stores (Mean: 1201 vs 807.2, p<0.001), poverty (Mean: 57.7% vs 45.5%, p<0.001), men (Mean: 46.7% vs 47.9%, p<0.001), households with TVs (Mean: 85.2% vs 81.9%, p<0.022), and also a higher MR from other causes than NCDs (Mean: 1005.7 vs 862.3, p<0.001).

**Fig 3:**
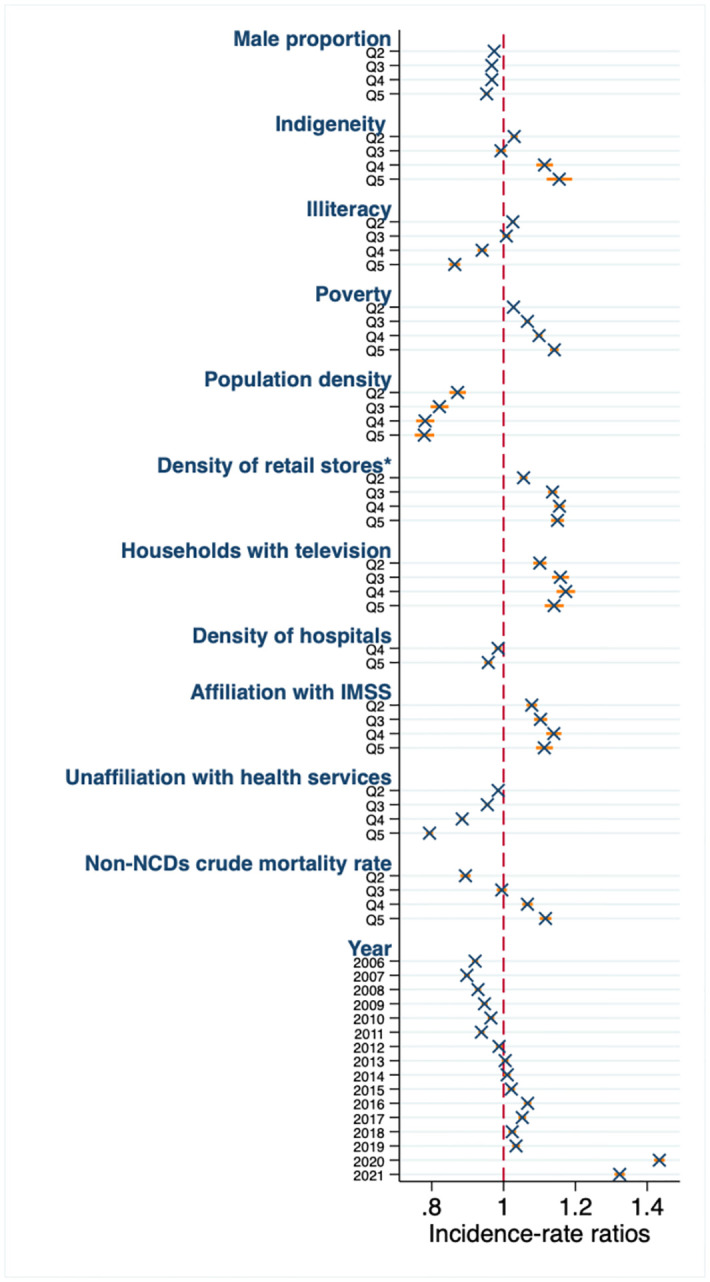
Mortality rate ratios of municipal factors per 100,000 Mexican adults and their association with the standardized mortality rate of non-communicable diseases from 2005 to 2021. Q: quintile. Q1: reference category. IMSS: Mexican Institute of Social Security. NCDs: non-communicable diseases. Estimates from binomial regression model adjusted by municipal structure of age, sex, and quintiles of the distribution of male proportion, indigeneity, illiteracy, poverty, population density, *density of ultra-processed food, alcohol & tobacco retail stores, households with television, density of hospitals, affiliated with IMSS, unaffiliated with health services, mortality rate from causes other than NCDs, year and state of the country. Predicted mortality rate was standardized by using the structure of age of 2015 for Mexican adults aged 20 years, from CONAPO.

Fig 3 displays the Incidence Rate Ratios (IRRs) for NCDs MR per 100,000 inhabitants by municipal factors. In each case, Q1 serves as the reference category. Municipal-level factors associated with higher NCDs MR included; indigeneity (IRR: 1.15 in Q5, p<0.001), poverty (IRR: 1.14 in Q5, p<0.001), the density of ultra-processed food, alcohol & tobacco retail stores (IRR: 1.15 in Q5, p<0.001), households with television (IRR: 1.14 in Q5, p<0.001), affiliation with IMSS (IRR: 1.11 in Q5, p<0.001) and the non-NCDs crude MR (IRR: 1.12 in Q5, p<0.001). Conversely, variables negatively associated with NCDs MR were; the male proportion (IRR: 0.95 in Q5, p<0.001), population density (IRR: 0.78 in Q5, p<0.001), hospitals density (IRR: 0.96 in Q5, p<0.001), unaffiliating with health services (IRR: 0.79 in Q5, p<0.001), and illiteracy (IRR: 0.86 in Q5, p<0.001). The expected MR decreased from 2006 to 2011 and increased from 2012 to 2021 (IRR: 0.92 in 2006 to 1.32 in 2021, p<0.001). The associations between medical consultations and hospital NCDs discharges did not show statistical significance and are not presented in Fig 3. The adjusted models for age groups 20–75 years were in line with the results from the model presented in Fig 3, which includes age groups beyond the life expectancy in Mexico. In S2 Table the model for the years 2005–2019 remained consistent when compared to the model for the years 2005–2021. Several municipal associations varied in the disaggregated models, although there was consistency in most of the estimates (see S2 Table).

## Discussion

The NCDs MR exhibited an upward trend in Mexican adults from 2005 to 2021. Our study highlighted significant geographic heterogeneity among municipalities across the country. This research is the first of its kind to establish NCDs mortality by municipality as it delves into an ecological small-area analysis to unveil how specific municipal characteristics, particularly social inequities, and ultra-processed food, alcohol & tobacco retail environment, play a significant role in the risk for NCDs mortality.

### Trends in NCDs mortality

The expected NCDs MR in Mexican adults increased over fifty percent from 2005 to 2021. Between 2015–2017, a trend emerged presenting an average annual decrease of two percentage points in the risk of NCDs mortality, persisting until the onset of the COVID-19 pandemic. This decline in NCDs mortality during this period could potentially be linked to government strategies focus on improving the quality of health care services in the previous years [28]. In 2020–2021, we observed a resurgence in MR compared to the preceding study period, which may be attributed to the advent of the COVID-19 pandemic.

The observed increase in MR across states and municipalities displayed marked heterogeneity, which could be attributed to the distribution of various ecologic health-related indicators [29]. A comprehensive study that assessed health-related sustainable development goals indicators in 1990–2017, including different measurements such as: socio-demographic, marginalization and concentration indexes, financial protection, poverty and health expenditure, revealed substantial disparities in performance among different states in Mexico [29]. For example, states such as Nuevo León and Sinaloa had a better progress towards the health-related indicators, and Oaxaca and Chiapas a worse performance. These disparities were related to socioeconomic factors including poverty and health expenditure [29].

### Municipal predictors of NCDs MR

As expected, mortality showed a direct correlation with age. However, from age 50, women had higher mortality than men. These findings aligned with a comprehensive meta-analysis, which attributed the higher NCD-related mortality among women to genetic, biological, gender disparities, cultural, and environmental factors [20]. Since the year 2000, countries in the Americas, excluding Mexico, experienced a decline in the CVD mortality among women; while Mexico experienced stagnation, a trend linked to its evolving socioeconomic development [21]. Consequently, gender disparities in the burden of NCDs has changed in favour of women which are less affected by tobacco and alcohol use but face a higher risk of physical inactivity [30].

Furthermore, our study revealed that municipalities with a higher proportion of indigenous populations had elevated NCDs mortality. Over the past two decades, MR among indigenous groups have been greater than those of non-indigenous populations, primarily attributed to NCDs [6]. While social and cultural determinants of indigenous health have been described, the heterogeneity in living environments [31] and the influence of commercial determinants [22] could offer additional explanations for our findings [22, 31]. A meta-analysis has suggested lower indigenous MR in urban compared to rural areas [22], likely attributable to the health advantages associated with urban living [22]. Regarding the commercial determinants of health, which involve private companies’ impact on public health goals, industries have been known to promote the consumption of UPP, alcohol, and tobacco [31]. Consequently, these unhealthy products have emerged as the primary drivers of health inequalities within indigenous population [31].

Our study also identified an unexpected association between a higher proportion of illiteracy and a lower NCDs mortality risk. These findings contrast with existing evidence linking lower education levels to decreased CVD mortality [23]. However, when analyzing NCDs mortality between 1990–2018, an increase in deaths was observed in countries with middle to low development countries while very-low development countries contributed the least to this mortality [19]. Authors attributed this trend to lower urbanization and dependence on local food in the least developed areas [19]. In this context, illiteracy may be linked to regional development, and a lower NCDs burden might be observed in least developed areas [19]. Another plausible explanation for the inverse correlation between illiteracy and NCDs mortality could be attributed to the positive trend in the life expectancy among illiteracy populations, while remained stable for non-illiterate populations [6, 33]. Further studies are needed to comprehensively analyze this relationship. Our results also exposed a lower NCDs mortality risk in municipalities with a higher proportion of population unaffiliated to health services. This finding could be attributed to undiagnosed morbidity and mortality resulting from NCDs. For example, after the implementation of health insurance programs in U.S.A. between 2007 and 2016, the authors observed an increase in NCDs prevalence trends from pre- to post-implementation programs [24].

In Mexico, despite improvements in social conditions since 2008, particularly in terms of healthcare access, there has been limited progress in population income [12, 28]. Poverty in this context, comprises factors that encompass the overall well-being of populations, including aspects like food security. This could result in insufficient fulfillment of essential needs, such as access to affordable and nutritious diets [28, 32]. Our results showed a notable association between municipalities with a higher proportion of poverty and an increased risk of NCDs mortality. However, within a food insecurity context, the population is more likely to turn to high-energy products, such as UPP, due to their affordability [32]. This propensity towards the consumption of such products increases their likelihood of obesity and, consequently, NCDs [32]. Regarding ultra-processed food, alcohol & tobacco retail environment, our results are consistent with a study where unhealthy food retail increased all-cause mortality, including NCDs [17]. The UPP contains sugars, saturated fats, sodium, and food additives linked to elevated morbidity and mortality from NCDs [33]. However, another study found limited evidence of associations between food retail and NCDs, possibly due to non-standardized measurements [4].

Population densities can be considered risk features for NCDs [14]. Despite that the high population density appears to be associated with higher NCDs mortality [14], we found that population density was inversely linked to NCDs mortality risk. Studies suggested that better infrastructure (for walking and cycling) in high-density cities might mitigate the density-mortality link [15]. Although infrastructure data at municipal-level is not publicly available in Mexico, a study [34] indicated that the sitting time (including watching television) and physical inactivity increased by more than 40% between 2006 and 2018. Higher NCDs mortality risk was found in our study for in municipalities where most of households have a television. Previous studies have linked prolonged television viewing to increased risk of NCDs and mortality [16].

Our investigation revealed that municipalities with higher hospital density had lower NCDs MR. In Mexico, hospitals are predominantly concentrated in urban areas, with only 3.3% located in rural regions [28]. This distribution of healthcare facilities raises a critical issue because individuals residing in rural areas face a higher mortality risk compared to those in urban, primarily due to healthcare inequalities and deficiencies in infrastructure and medical personnel [12, 35]. Thus, the observed protective effect of hospital density against NCDs MR underscores the persistent rural-urban health disparities that exists within the country. Interestingly, our research uncovers a paradoxical finding in relation to public healthcare services and NCDs mortality. We observed that municipalities with high proportion of affiliation with IMSS had a higher risk of NCDs mortality, even in the model for the period from years 2005 to 2019. IMSS is the largest public healthcare provider in the country, covering approximately 38% of adults, however also faces challenges [36], particularly during COVID pandemic. According to reports [6] in 2021 42.0% (469,542) of deaths in Mexico occurred in public and private hospitals, with 43% of these occurring within the IMSS, and the remainder in other healthcare facilities [6]. Consequently, our findings regarding mortality and IMSS affiliation must be interpreted cautiously recognizing the complex and multifaceted landscape of healthcare services in Mexico.

Estimating age-adjusted and standardized MR offers the advantage of reducing the ’volatility’ in the estimations for municipalities with very small populations. In such cases, we often observe mortality rates in the thousands (e.g., in 2021 municipality Santo Domingo Tlatayápam from Oaxaca, with 4 cases and 102 inhabitants, had a crude MR = 3,922 per 100,000 inhabitants vs an estimated MR = 513.5, CI 95% 487.6–540.8) where a single death can exert a significant impact on the metrics due to the very small population denominator. Therefore, relying solely on crude mortality rates to target preventive and treatment efforts in these municipalities may not necessarily pinpoint the most urgent areas for intervention. Consequently, this article provides insights into identifying priority locations across the country that require targeted interventions to prevent mortality cases related to NCDs and outlines the characteristics of these municipalities.

### Strengths and limitations

Our study is novel in using a municipal approach to describe NCDs mortality predictors in Mexico between 2005 and 2021. The methodology can be applied to other diseases. Our study also provides a broader understanding of NCDs mortality heterogeneity throughout the country, providing relevant knowledge on the specific health needs of each state and municipality. We analyzed extensive data across years integrating a relevant number of observations and showing robust and consistent results. Limitations include the ecological design lacking temporal criteria; therefore, there is no causal inference between the risk factors and NCDs mortality. Caution against ecological fallacy suggests interpreting results as group-based instead of individual. The precision of our estimations depended on the quality and the study covariates representativeness. Moreover, since population size was relatively small for some municipalities, the observed rate could differ from the expected due to the random data variation. Finally, measurement errors are usually greater in smaller areas than in large areas, so the estimates’ reliability is more precise in the former. Nevertheless, the covariables in this study at the municipal level produced representative estimations with predictive strength.

## Conclusion

The NCDs MR in Mexican adults increased from 2005 to 2021 and varied significantly at the municipal-level. Our results suggest that the municipal predictors of the NCDs mortality risk were related to inequities in sociodemographic factors, household characteristics, access to healthcare services and density of ultra-processed food, alcohol & tobacco retail stores. Mexican adults with NCDs require further targeted public policies and social programs to enhance their life prognosis. Our study can inform future public policies aimed at reducing inequalities across states and municipalities.

## Supporting information

S1 Table Top 100 Mexican municipalities with the highest standardized mortality rate of non-communicable diseases per 100,000 inhabitants, 2005–2021. (XLSX)

S2 Table Mortality rate ratios of municipal factors per 100,000 Mexican adults and their association with the standardized mortality rate of disaggregated non-communicable diseases from 2005 to 2021. (XLSX)

S1 Data (CSV)

S2 Data (CSV)

## References

[1] World Health Organization. 2022 [Accessed October 04, 2022]. https://www.who.int.

[2] Agudelo-Botero M, Valdez-Ortiz R, Giraldo-Rodriguez L, Gonzalez-Robledo MC, Mino-Leon D, Rosales-Herrera MF, et al. Overview of the burden of chronic kidney disease in Mexico: secondary data analysis based on the Global Burden of Disease Study 2017. BMJ Open. 2020;10(3):e035285. doi: 10.1136/bmjopen-2019-035285 32213523 PMC7170614

[3] GBD 2019 Diseases and Injuries Collaborators. Global burden of 369 diseases and injuries in 204 countries and territories, 1990–2019: a systematic analysis for the Global Burden of Disease Study 2019. Lancet. 2020;396(10258):1204–22. doi: 10.1016/S0140-6736(20)30925-9 33069326 PMC7567026

[4] de Albuquerque FM, Pessoa MC, De Santis Filgueiras M, Gardone DS, de Novaes JF. Retail food outlets and metabolic syndrome: a systematic review of longitudinal studies. Nutr Rev. 2022;80(6):1599–618. doi: 10.1093/nutrit/nuab111 35182145

[5] Contreras-Manzano A, Guerrero-Lopez CM, Aguerrebere M, Sedas AC, Lamadrid-Figueroa H. Municipality-Level Predictors of COVID-19 Mortality in Mexico: A Cautionary Tale. Disaster Med Public Health Prep. 2022;16(4):1384–92. doi: 10.1017/dmp.2020.485 33731243 PMC7985638

[6] National Institute of Statistics and Geography. 2022 [Accessed October 01, 2022]. https://www.inegi.org.mx.

[7] Checchi F, Testa A, Gimma A, Koum-Besson E, Warsame A. A method for small-area estimation of population mortality in settings affected by crises. Popul Health Metr. 2022;20(1):4. doi: 10.1186/s12963-022-00283-6 35016675 PMC8751462

[8] Aceves B, Ingram M, Nieto C, de Zapien JG, Rosales C. Non-communicable disease prevention in Mexico: policies, programs and regulations. Health Promot Int. 2020;35(2):409–21. doi: 10.1093/heapro/daz029 31006024 PMC7313413

[9] Barker LE, Thompson TJ, Kirtland KA, Boyle JP, Geiss LS, McCauley MM, et al. Bayesian Small Area Estimates of Diabetes Incidence by United States County, 2009. J Data Sci. 2013;11(1):269–80. 26279666 PMC4537395

[10] Center for the Study of Sustainable Urban and Regional Development. 2019 [Accessed October 20, 2022.]. https://cedrus-unam.blogspot.com/2019/04/datos.html.

[11] General Secretariat of the National Population Council. 2023 [Accesed March 20, 2024.]. https://datos.gob.mx/busca/organization/conapo.

[12] National Council for the Evaluation of Social Development Policy. 2022 [Accessed October 20, 2022.]. https://www.coneval.org.mx/Medicion/Paginas/Pobreza-municipio-2010-2020.aspx.

[13] Ministry of Health of Mexico. Deaths, Open Data—Dirección General de Información en Salud. 2022 [Accessed October 30, 2022.]. http://www.dgis.salud.gob.mx/contenidos/basesdedatos/da_defunciones_gobmx.html.

[14] Carnegie ER, Inglis G, Taylor A, Bak-Klimek A, Okoye O. Is Population Density Associated with Non-Communicable Disease in Western Developed Countries? A Systematic Review. Int J Environ Res Public Health. 2022;19(5). doi: 10.3390/ijerph19052638 35270337 PMC8910328

[15] Beenackers MA, Oude Groeniger J, Kamphuis CBM, Van Lenthe FJ. Urban population density and mortality in a compact Dutch city: 23-year follow-up of the Dutch GLOBE study. Health Place. 2018;53:79–85. doi: 10.1016/j.healthplace.2018.06.010 30056264

[16] Grontved A, Hu FB. Television viewing and risk of type 2 diabetes, cardiovascular disease, and all-cause mortality: a meta-analysis. JAMA. 2011;305(23):2448–55. doi: 10.1001/jama.2011.812 21673296 PMC4324728

[17] Lovasi GS, Johnson NJ, Altekruse SF, Hirsch JA, Moore KA, Brown JR, et al. Healthy food retail availability and cardiovascular mortality in the United States: a cohort study. BMJ Open. 2021;11(7):e048390. doi: 10.1136/bmjopen-2020-048390 34244272 PMC8273445

[18] Ríos V, Denova-Gutiérrez E, Barquera S. Association between living in municipalities with high crowding conditions and poverty and mortality from COVID-19 in Mexico. PLoS One. 2022;17(2):15. doi: 10.1371/journal.pone.0264137 35192660 PMC8863291

[19] Manderson L, Jewett S. Risk, lifestyle and non-communicable diseases of poverty. Global Health. 2023;19(1):13. doi: 10.1186/s12992-023-00914-z 36864476 PMC9978269

[20] Wang Y, O’Neil A, Jiao Y. Sex differences in the association between diabetes and risk of cardiovascular disease, cancer, and all-cause and cause-specific mortality: a systematic review and meta-analysis of 5,162,654 participants. BMC Med. 2019;(17):18. doi: 10.1186/s12916-019-1355-0 31296205 PMC6625042

[21] Lanas F, Soto A. Trends in Mortality from Ischemic Heart Disease in the Region of the Americas, 2000–2019. Glob Heart. 2022;17(1):53. doi: 10.5334/gh.1144 36051321 PMC9374038

[22] Carson E, Sharmin S, Maier AB, Meij JJ. Comparing indigenous mortality across urban, rural and very remote areas: a systematic review and meta-analysis. Int Health. 2018;10(4):219–27. doi: 10.1093/inthealth/ihy021 29617891

[23] Pednekar MS, Gupta R, Gupta PC. Illiteracy, low educational status, and cardiovascular mortality in India. BMC Public Health. 2011;11:567. doi: 10.1186/1471-2458-11-567 21756367 PMC3160988

[24] Musonge-Effoe JE, Alema-Mensah E, Effoe VS, Akinnawo F, Caplan L. The association between health care coverage and prevalence of cardiovascular diseases and diabetes over a 10-year period. Prev Med. 2020;132:105983. doi: 10.1016/j.ypmed.2020.105983 31954838

[25] Hernández-F M, Figueroa JL, Colchero MA. Association between density of stores and purchases of ultra-processed food and sugar-sweetened beverages in Mexico. Health Place 2021;(68):8. doi: 10.1016/j.healthplace.2021.102528 33662788

[26] Kim JH. Multicollinearity and misleading statistical results. Korean J Anesthesiol. 2019;72(6):558–69. doi: 10.4097/kja.19087 31304696 PMC6900425

[27] Antonio-Villa NE, Bello-Chavolla OY, Fermin-Martinez CA, Aburto JM, Fernandez-Chirino L, Ramirez-Garcia D, et al. Socio-demographic inequalities and excess non-COVID-19 mortality during the COVID-19 pandemic: a data-driven analysis of 1 069 174 death certificates in Mexico. Int J Epidemiol. 2022;51(6):1711–21. doi: 10.1093/ije/dyac184 36174226 PMC9619535

[28] Gonzalez Block MA, Reyes Morales H, Hurtado LC, Balandran A, Mendez E. Mexico: Health System Review. Health Syst Transit. 2020;22(2):1–222. 33527902

[29] Gutierrez JP, Agudelo-Botero M, Garcia-Saiso S, Zepeda-Tena C, Davila-Cervantes CA, Gonzalez-Robledo MC, et al. Advances and challenges on the path toward the SDGs: subnational inequalities in Mexico, 1990–2017. BMJ Glob Health. 2020;5(10). doi: 10.1136/bmjgh-2020-002382 33122296 PMC7597504

[30] Guerrero-Lopez CM, Servan-Mori E, Miranda JJ, Jan S, Orozco-Nunez E, Downey L, et al. Burden of non-communicable diseases and behavioural risk factors in Mexico: Trends and gender observational analysis. J Glob Health. 2023;13:04054. doi: 10.7189/jogh.13.04054 37326368 PMC10274265

[31] Crocetti AC, Cubillo Larrakia B, Lock Ngiyampaa M, Walker Yorta Yorta T, Hill Torres Strait Islander K, Mitchell Mununjali F, et al. The commercial determinants of Indigenous health and well-being: a systematic scoping review. BMJ Glob Health. 2022;7(11). doi: 10.1136/bmjgh-2022-010366 36319033 PMC9628540

[32] Carvajal-Aldaz D, Cucalon G, Ordonez C. Food insecurity as a risk factor for obesity: A review. Front Nutr. 2022;9:1012734. doi: 10.3389/fnut.2022.1012734 36225872 PMC9549066

[33] Dehghan M, Mente A, Rangarajan S, Mohan V, Swaminathan S, Avezum A, et al. Prospective Urban Rural Epidemiology (PURE) study investigators. Ultra-processed foods and mortality: analysis from the Prospective Urban and Rural Epidemiology study. Am J Clin Nutr. 2023;117(1):55–63. doi: 10.1016/j.ajcnut.2022.10.014 36789944

[34] Medina C, Jauregui A, Hernandez C, Shamah T, Barquera S. Physical inactivity and sitting time prevalence and trends in Mexican adults. Results from three national surveys. PLoS One. 2021;16(7):e0253137. doi: 10.1371/journal.pone.0253137 34214109 PMC8253416

[35] Rivera-Hernandez M, Ferdows NB, Kumar A. The Impact of the COVID-19 Epidemic on Older Adults in Rural and Urban Areas in Mexico. J Gerontol B Psychol Sci Soc Sci. 2021;76(7):e268–e74. doi: 10.1093/geronb/gbaa227 33367752 PMC7798580

[36] Gonzalez Block MA, Diaz Portillo SP, Morales HR, Rodriguez Saldana J, Gutierrez Calderon E. Diabetes care innovation in the Mexican Institute for Social Insurance: Scaling up the preventive chronic disease care model to address critical coverage constraints. Prim Care Diabetes. 2021;15(2):314–22. doi: 10.1016/j.pcd.2020.10.012 33199194

